# Development of Active Packaging Based on Agar-Agar Incorporated with Bacteriocin of *Lactobacillus sakei*

**DOI:** 10.3390/biom11121869

**Published:** 2021-12-13

**Authors:** Camila Ramão Contessa, Nathieli Bastos de Souza, Guilherme Battú Gonçalo, Catarina Motta de Moura, Gabriela Silveira da Rosa, Caroline Costa Moraes

**Affiliations:** 1 Program in Materials Science and Engineering, Federal University of Pampa, 1650, Maria Anunciação Gomes Godoy Avenue, Bagé 96413-170, RS, Brazil; camilaramao@hotmail.com (C.R.C.); gabrielarosa@unipampa.edu.br (G.S.d.R.); 2 Program in Food Engineering and Science, School of Chemistry and Food, Campus Carreiros, Federal University of Rio Grande, Av. Itália Km 08, Rio Grande CEP 96203-900, RS, Brazil; nathieli.souza.1995@gmail.com; 3 Food Engineering Course, Campus Bagé, Federal University of Pampa, Av. Maria Anunciação Gomes Godoy, 1650, Bagé CEP 96413-170, RS, Brazil; guibattu@hotmail.com (G.B.G.); catarinamoura@unipampa.edu.br (C.M.d.M.)

**Keywords:** bioconservation, pathogens, lactic acid bacteria

## Abstract

In the search for new biodegradable materials and greater microbiological safety and stability of perishable food products, this study aimed to develop a bioplastic antibacterial film incorporating bacteriocin for application in commercial curd cheese and monitoring of microbiological stability. Films with good handling characteristics as well as physical, barrier, and mechanical properties were obtained. Regarding the antibacterial activity, the microbial reduction was demonstrated in a food matrix, obtaining a reduction of 3 logarithmic cycles for the group of coagulase positive staphylococci and from 1100 to <3.00 MPN/g in the analysis of thermotolerant coliforms. Therefore, the film presented food barrier characteristics with the external environment and adequate migration of the antibacterial compound to the product, contributing to the reduction of contamination of a food with high initial microbial load.

## 1. Introduction

Bioplastic films are materials synthesized from natural sources which present much higher degradation than conventional films of petrochemical origin [1]. Structurally, they present dynamic materials with the most diverse characteristics, related to the techniques of preparation for the materials used [2]. Therefore, they present a wide range of applications, and when developed for application in the food industry, they are susceptible to the incorporation of antimicrobial compounds. Together with the reduction of the environmental impact, they act in the increasement of the microbiological stability of the foods [3].

The growing demand of the population for natural foods free of synthetic chemical additives implies the development of alternative technologies for food conservation, mainly for combating pathogenic microorganisms, which have the same effectiveness as conventional methods. This demand has led to the use of natural antimicrobials from plant, animal, or microbial sources [4].

The use of bacteriocins provides a potential solution, especially those produced by lactic acid bacteria, which are of great interest to the food industry. The bacteria belonging to this group, are already used as starter cultures in fermented products and many have the status “generally recognized as safe” (GRAS), granted by the Food and Drug Administration (FDA) [5]. These bacteriocins are still non-toxic to cells of the human organism; however, they present effects against several food spoilage microorganisms and pathogenic bacteria [5,6].

Perishable products present nutritional characteristics favorable to microbial growth even in refrigeration conditions, due to their high humidity. According to Marsh and Bugusu [7], this sector is the one that most contributes with the generation of residues, representing around 50% in weight of the total packages sold. Thus, in order to promote the microbiological safety of these products and, at the same time, contribute to the reduction of environmental impacts generated by the food industry, the present work is justified. It aims to develop a bioplastic film synthesized from natural compounds such as agar-agar and a bacteriocin from *Lactobacillus sakei* isolated from a food matrix as well as its application in a food product of high perishability (ricotta) and the monitoring of its microbiological stability.

## 2. Methodology

### 2.1. Bacteriocin Production by Lactobacillus sakei

A strain of *Lactobacillus sakei,* isolated from Italian salami, was used to obtain bacteriocin by the research group of the Microbiology and Food Toxicology Laboratory of the UNIPAMPA Bagé campus, and identified by the Pluridisciplinary Center for Chemical, Biological and Agricultural Research of the State University of Campinas (UNICAMP). The microorganism was cultivated in liquid medium Man, Rogosa and Sharpe (MRS), at 32 °C for 24 h and orbital agitation of 150 rpm. The removal of the cells occurred by refrigerated centrifugation at 5500 rpm for 15 min. After this process, the supernatant was analyzed, and the precipitate discarded. The supernatant without any treatment was called cell-free extract (CFE).

### 2.2. Production and Characterization of Active Bioplastic Films Incorporated of Bacteriocin

The films were developed using the casting technique with Agar-Agar (Himedia, WF, Pelotas-RS, Brazil) as a biopolymer, which comes from a mixture of two polysaccharides (agarose and agaropectin). In order to improve the flexibility and mechanical resistance of the film, glycerol (Alphatec, WF, Pelotas-RS, Brazil)) was used as a plasticizer. To provide the film with the active characteristic, CFE was used in the liquid state at concentrations of 0%, 25%, 50%, and 75% of the filmogenic solution. A control film used as parameter was developed without the addition of CFE, using only solvent and the same proportions of plasticizer and polymeric matrix. The films were named AA0, A25, A50, and A75, corresponding to the following films: control without addition of extract, 25% of extract, 50% of extract, and 75% of extract, respectively.

#### 2.2.1. Thickness

With the support of a digital pachymeter (DIGIMESS 0.01 mm), the measurements were taken at ten different positions of the film, detecting the mean thickness of the different samples of the biodegradable films produced [8].

#### 2.2.2. Water Vapor Permeability (WVP)

The permeability was determined gravimetrically by the standard method of the American Society for Testing and Materials (ASTM) E96/E96M—14 [9]. The films were applied in capsules containing calcium chloride (CaCl_2_), and the set was conditioned in a chamber with relative humidity of 50%. The moisture gain was determined over 7 days and the WVP was calculated from Equation (1).

(1) $$P_{go}\;=\;\frac{M_{p\;}L}{tA\Delta P}$$

where $P_{go}$ is the water vapor permeability ($\frac{kg}{Pa.s.m}$), $M_{p\;}$ is the mass of moisture absorbed (g), L is the thickness of the film (mm), *A* is the area of the exposed surface of the film (mm), and ΔP is the partial pressure difference through the film.

#### 2.2.3. Solubility in Water (WS)

Film samples were uniformly cut in 2 cm diameter circles and dried at 105 °C for 24 h to determine their initial dry weight. Then, the samples were placed in recipients with 50 mL of distilled water and kept under orbital agitation of 175 rpm for 24 h at a temperature of 25 °C. The undissolved films were filtered and dried at 105 °C for 24 h to determine their final dry weight. The solubility of the films was expressed as a function of the initial and final dried mass, from the use of Equation (2) [10].

(2) $$WS=\;\frac{m_{i}-m_{f}}{100}\times 100$$

where *WS* is the solubility in water, *mi* (g) is the initial dried mass, and *mf* (g) is the final dried mass.

#### 2.2.4. Mechanical Properties

The tensile and elongation at break point were performed using texturometer (Stable Micro System TA.XTplus, https://www.stablemicrosystems.com/TAXTplus.html accessed on 16 February 2021) according to standard method D 882—12 (ASTM) [11]. The films were cut into 25 mm wide and 100 mm long specimens. Samples were clamped and deformed under tensile loading using a 50 N load cell with an initial grip separation of 25 mm and a crosshead speed of 50 mm min^−1^. The tensile stress or tensile strength was obtained from Equation (3), followed by the calculation of elongation at rupture represented by Equation (4).

(3) $$TR=\frac{F_{m}}{A}$$

where *TR* is the ultimate tensile strength (*MPa*), *Fm* is the maximum force at break point (*N*), and *A* is the cross-sectional area (m^2^).

(4) $$E=\frac{d_{r}}{d_{i}}\times 100$$

where *E* is represented by the elongation (%), *d_r_* is the distance at the break point (cm), and *d_i_* is the initial separation distance (cm).

#### 2.2.5. Microbiological Characterization of Films

The antibacterial activity of the films was evaluated through the adapted disc-diffusion methodology described by NCCLS [12]. A 0.1 mL aliquot of microbial culture (*Escherichia coli*, *Staphylococcus aureus*, *Salmonella enteritidis*, and *Listeria monocytogenes*) was added, standardized with approximately 1.5 × 10^8^ CFU/mL, and Mueller Hinton agar was added, and after solidification of the agar, 6 mm of film diameter (0%, 25%, 50%, and 75% of CFE) were added on the surface, and the plates were incubated at 35 °C/24 h. The inhibition halos represented by a clear zone without microbial development were measured with a digital pachymeter.

### 2.3. Application of Active Bioplastic Film in Curd Cheese and Monitoring of Microbiological Stability

**Figure 1:**
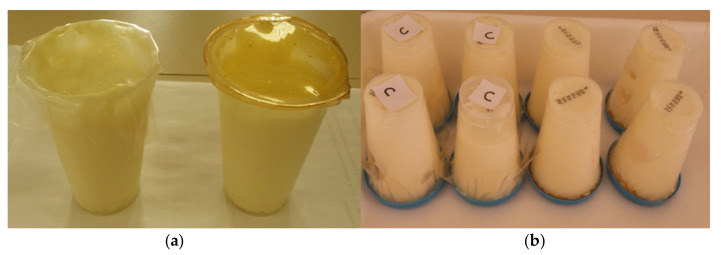
Application of active bioplastic film on curd cheese. (**a**) Replacement of the aluminum membrane for the control film and extract; (**b**) Inverted product storage so that it comes in direct contact with the film.

To simulate the use of the film applied to food, part of the films (with and without the bacteriocin extract) were adhered to the traditional curd packaging, substituting for the aluminum membrane traditionally used in this kind of product, according to Figure 1.

This way, thermotolerant coliforms and coagulase positive staphylococci were analyzed on the 7th, 21st, and 28th accompanying day, and during this time, the product was stored inverted so that it would be in contact with the films at refrigeration temperature (Figure 1).

An intentional contamination with 10^8^ CFU/mL of *Staphylococcus aureus* and *Escherichia coli*, separately to a curd cheese sample, was also made to validate the results obtained.

The analyzed microorganisms were thermotolerant coliforms and positive coagulase staphylococci performed by the traditional method of multiple tubes, in accordance with the American Public Health Association (APHA), and direct plate count method of APHA [13].

### 2.4. Statistical Analysis of Results

All results were submitted to a mean comparison test, with data that met the assumptions of independence, normality, and homoscedasticity. In order to verify the existence of significant differences between the conditions studied. The post hoc Tukey test were applied with a 95% confidence level (*p* < 0.05).

## 3. Results and Discussions

From the values obtained in Table 1 for the results of the mechanical, barrier, and physical properties for the elaborated films, it is observed that the control film and A25 did not present a significant difference in thickness, which were different from A50 and A75. As the extract concentration increases, the thickness is increased, which can be explained by the presence of suspended solids in the extract; this same behavior was also observed by [14,15].

It is observed that the increase in the amount of extract was inversely proportional to the WVP (water vapor permeability), a promising result for the performed application since the interaction with the external environment interferes with the sensory stability and commercial sterility of the food. The same behavior was observed by [16] that when adding clay and nanocomposites of agar to agar-based film formulations had a reduction in water vapor permeability of 2.22 ± 0.19 to 1.07 ± 0.05 g m/m^2^ s Pa, as observed in [17] and [18] for chitosan and whey-based films, respectively.

The results indicate that the addition of the extract in the composition of the films significantly increased the solubility. Similar behavior was found by [19] for agar-based films formulated with k-carrageenan and konjac glucomannan. The film containing the three components showed a water solubility of 100%, and the film without the konjac fiber showed a solubility of 14.7%. However, the film produced in this study can still be used in a food matrix with a high moisture content, as it is not completely soluble when in contact with water.

Regarding mechanical properties such as elongation and tensile strength, the results for elongation were directly proportional to the increase of extract, that is, the control film had the lowest elongation and the film with the highest amount of extract (A75) had the highest elongation. Frequently films with a high elongation present a lower need of applied force for the rupture, behavior that was observed in this study. The elongation values were higher and the tensile strength values were lower than those reported by [18,20] in agar films reinforced with nanocellulose and microcrystalline cellulose and in whey protein film with organophilic bentonite.

It is noted that the properties analyzed in this study are similar to the results in the literature, they used improving compounds, such as clay and nanocomposites, thus highlighting the results found.

Table 2 presents the results of the analysis of the antimicrobial effect of the films, where can it be observed the halos of inhibition formed by the films developed against *Escherichia coli*, *Staphylococcus aureus*, *Salmonella enteritidis*, and *Listeria monocytogenes* are observed.

All the films, regardless of the amount of bacteriocin added, demonstrated the capacity of inhibition on any of the evaluated microorganisms, whereas when bacteriocin was not added in the film formulation, no inhibition was found. For *Escherichia coli*, it was possible to observe that the increase in the percentage of extract in the films was directly related to the increase in the halos of inhibition. The statistical analysis indicated that for *Escherichia coli*, all the films differed among themselves, while for *Staphylococcus aureus*, the obtained halos did not present significant difference in relation to the developed films. The behavior towards *Salmonella* indicated that A25 and A50 films are the same among themselves, and for *Listeria monocytogenes*, A50 film did not present a significant difference in relation to A25 and A75 in relation to the obtained inhibition halos. The AA0 showed no signs of inhibition against any microorganism, which was already expected in view of the absence of bacteriocin extract in its formulation.

Shahbazi, Shavisi, and Mohebi [21] obtained, in their studies, a reduction in the count of *Staphylococcus aureus* from 4.30 to 3.34 and 2.05 when using 250 and 500 IU/g of nisin, respectively. Thus, it is noted that the values obtained for this work are similar to those in the literature, where the extract was used without any purification through the analyzed conditions.

Regarding the active packaging and the incorporation of bacteriocins the literature is very restricted. There are studies of application only of commercial bacteriocins, such as nisin and pediocin, due to its GRAS approval by the US Food and Drug Administration (FDA) [22]. Meira et al. [23] produced films with a polymeric matrix of corn starch embedded in nisin and pediocin. Santiago-Silva et al. [24] evaluated the antimicrobial effect of films with a cellulose matrix and incorporated with pediocin in the preservation of sliced ham. Xiong et al. [25] evaluated the storage of fresh pork with edible chitosane–gelatine-based coating incorporated nisin extract with grape seed.

Nisin and pediocin showed antimicrobial activities against a wide variety of Gram-positive bacteria, and there are reports of using as natural bioconservatives in meat and dairy products; however, their spectrum against Gram-negative microorganisms is deficient [26]. Thus, the investigation for bacteriocins with broad spectrum of action for both Gram-positive and Gram-negative microorganisms is promising.

Therefore, the A25 film was chosen for application in curd cheese because it has less extract, thus reducing future expenses for a possible increase in scale, besides presenting a low solubility, taking into account that the application product has high humidity, it also presented the best mechanical properties, essential for the handling of the film and packaging of the product besides presenting acceptable microbiological characteristics.

### Microbiological Monitoring of the Packed Curd Cheese with the Active Film

The A25 film was added to the curd cheese, replacing the commercially present aluminum cover. Table 3 presents the values obtained for thermotolerant coliforms with the analyses performed.

It is observed that after the storage, a reduction in the count was obtained, including the control film, due to injuries caused by the cold, because in [27], in is said that the cold is one of the methods most used for the conservation of food because it acts in inhibiting or delaying the multiplication of microorganisms. However, it is noticeable that the A25 film contributed to a greater reduction in the microbial load since it showed a reduction of 92.8% compared to AA0 with the same time for storage.

For the validation of results, the curd cheese was intentionally contaminated with 10^8^ CFU/mL of *Staphylococcus aureus* and *Escherichia coli* in order to validate the analysis, which remained with the same behavior, presenting a great reduction in the count when comparing the A25 film with the control film, resulting in a count of <3.0 ± 0.00 MPN/g for A25 and 28 ± 0.01 MPN/g for the AA0 control film for 7 days of storage.

Botelho et al. [28] found, in their analyses, that 14.3% of the analyzed ricotta was not in conformity with the legislation. Given the above, the importance of using this type of active packaging is noted in order to reduce the incidence of these high contaminations. It can be seen that the bacteriocin embedded film studied in the present work has reduced from 1100 to less than 3 MPN/g, presenting itself as a great alternative for use in products of this origin. The results for positive coagulase staphylococci can be seen in Table 4.

The reduction in the CFU/g count of the A25 film when compared to AA0 in all the analyses is noticeable, in the same way it occurred in the confirmation of the results, presenting a contamination of 7.2 × 10^7^ CFU/g for the AA0 control film and 6.9 × 10^4^ ± 0.1 × 10^7^ (CFU/g) for the A25 film, a very significant result since a reduction of 3 logarithmic cycles was obtained.

*Staphylococcus aureus* strains have a toxicity that is observed by their capacity to coagulate blood plasma, made possible by the presence of the coagulase enzyme, thus differentiating itself from other microorganisms present in the staphylococcal group [29]. Thus, all the microorganisms analyzed were tested for the confirmation of positive coagulase staphylococci strains.

Abdollahzadeh, Hosseini, and Fooladi [30] obtained an inhibition halo of 11 mm against *Staphylococcus aureus* when using 100 UA of nisin nanoparticles in agar films and 11 mm when using 5 mg of ZnO in agar films. Capelezzo et al. [31] obtained a 99.9% reduction of *Staphylococcus aureus* colonies when using polymeric films plus zinc oxide nanoparticles.

The concern with this microorganism is due to its great involvement in food contamination outbreaks; since it is present in human airways, the contamination of food handled without the proper hygienic sanitary conditions is very frequent. Staphylococcal food poisoning is one of the most common and recurrent food diseases, since the first report of a disease originating from *Staphylococcus aureus* occurred in 1884 due to the consumption of a contaminated cheese [32].

Due to the great incidence of contamination by *Staphylococcus aureus*, caused due to inadequate hygiene and handling practices, this study is of great value since it presents an alternative for the reduction of these numbers from the application of an antimicrobial film that has efficiency in reducing the development of this microorganism.

## 4. Conclusions

The developed films presented adequate mechanical properties for handling, requiring strength between 1.67 to 4.70 MPa for breaking and presenting elongation of 25.9% to 94.1%. The solubility varied from 23.80% to 77.18% and the water vapor permeability from 0.11 to 1.06 × 10^−7^ g∙m^−1^∙Pa^−1^∙s^−1^. All the films with incorporated extract presented antibacterial property, with inhibition halos varying from 7.32 to 9.33 mm in diameter. The film chosen for the application was the A25 formulation because it presents the lowest solubility and the higher strength at break.

It was possible to observe the efficiency of bacteriocin when incorporated in a filmogenic matrix, presenting a reduction of 3 logarithmic cycles for the curd stored with the incorporated bacteriocin film, the contamination by thermotolerant coliforms was reduced from 1100 to <3.00 MPN/g.

Thus, through the results found with the use of this compound, it is possible to observe that the bacteriocin produced by the studied *Lactobacillus sakei* has great potential for application in films with antibacterial action. It was found that films based on agar-agar and incorporated bacteriocin can be used as active packaging, since they presented adequate characteristics for handling and also for conservation. When applied to a food matrix, it contributed to the release of antibacterial compound and consequently increased the microbiological stability of the analyzed product, drastically reducing the contamination of a food with high initial microbial load.

## Figures and Tables

**Table 1:** Characteristics of films.

Film	Thickness (mm)	WVP (× 10^−7^ g m^−1^ Pa^−1^ s^−1^)	Solubility (%)	TR (MPa)	E (%)
AA0	0.07 ± 0.01 ^a^	1.06 ± 1.07 ^a^	23.80 ± 0.42 ^a^	3.25 ± 0.39 ^a^	25.9 ± 0.40 ^b^
A25	0.06 ± 0.01 ^a^	0.65 ± 0.09 ^b^	43.24 ± 0.37 ^b^	4.70 ± 0.42 ^c^	48.24 ± 0.39 ^a^
A50	0.24 ± 0.02 ^b^	0.16 ± 0.19 ^c^	59.43 ± 0.45 ^c^	1.67 ± 0.23 ^b^	44.42 ± 0.41 ^a^
A75	0.29 ± 0.00 ^c^	0.11 ± 0.01 ^d^	77.18 ± 0.36 ^d^	2.20 ± 0.35 ^ab^	94.1 ± 2.54 ^c^

The results are presented as mean ± standard deviation. Equivalent letters ^a, b, c, d^ in the column represent values without significant differences (*p* < 0.05) in Tukey test. WVP—water vapor permeability, TR—tensile strength, E—elongation.

**Table 2:** Diameters of the inhibition halos formed by the films.

	Inhibition Halos (mm)			
	*Escherichia coli*	*Staphylococcus aureus*	*Salmonella enteritidis*	*Listeria monocytogenes*
AA0	There were no inhibition halos			
A25	7.34 ± 0.01 ^a^	8.37 ± 0.02 ^a^	7.32 ± 0.03 ^a^	7.98 ± 0.32 ^a^
A50	8.69 ± 0.01 ^b^	8.41 ± 0.15 ^a^	7.32 ± 0.13 ^a^	8.97 ± 0.42 ^ab^
A75	9.33 ± 0.02 ^c^	8.52 ± 0.03 ^a^	8.05 ± 0.26 ^b^	9.02 ± 0.30 ^b^

The results are presented as mean ± standard deviation. Equivalent letters ^a, b, c^ in the column represent inhibition values without significant differences with *p* < 0.05 in Tukey test.

**Table 3:** Count of thermotolerant coliforms (MPN/g).

Days of Analysis	AA0	A25
Time 0	1100 ± 0.00 ^a^	
7th Day	210 ± 0.00 ^bA^	15 ± 0.00 ^bB^
21st Day	43 ± 0.00 ^cA^	9.2 ± 0.00 ^cB^
28th Day	39 ± 0.00 ^dA^	7 ± 0.00 ^dB^

Values are presented as mean ± standard deviation. Different lowercase letters ^a, b, c, d^ in the same column indicate significant differences (*p* < 0.05). Different capital letters ^A, B^ on the same line indicate significant differences (*p* < 0.05).

**Table 4:** Coagulase positive staphylococcal contamination (CFU/g).

Days of Analysis	AA0	A25
Time 0	5 × 10^5^ CFU/g ± 1.2 × 10^5 a^	
7th Day	1.1 × 10^5^ ± 1.0 × 10^5 bA^	1.1 × 10^4^ ± 1.3 × 10^4 bB^
21st Day	2.0 × 10^4^ ± 1.2 × 10^4 cA^	1.3 × 10^4^ ± 1.1 × 10^4 bB^
28 Day	1.2 × 10^4^ ± 1.1 × 10^4 dA^	1.7 × 10^3^ ± 1.1 × 10^4 cB^

Values are presented as mean ± standard deviation. Different lowercase letters ^a, b, c, d^ in the same column indicate significant differences (*p* < 0.05). Different capital letters ^A, B^ on the same line indicate significant differences (*p* < 0.05).

## References

[1] Peelman N., Ragaert P., Meulenaer B., Adons D., Peeters R., Cardon L., Van Impe F., Devlieghere F. (2013). Application of bioplastics for food packaging. Trends Food Sci. Technol..

[2] Mateescu A., Wang Y., Dostalek J., Jonas U. (2012). Thin Hydrogel Films for Optical Biosensor Applications. Membranes.

[3] Latos-Brozio M., Masek A. (2020). The application of natural food colorants as indicator substances in intelligent biodegradable packaging materials. Food Chem. Toxicol..

[4] Zacharof M.P., Lovitt R.W. (2012). Bacteriocins Produced by Lactic Acid Bacteria a Review Article. APCBEE Procedia.

[5] Gálvez A., Abriouel H., López R.L., Omar N.B. (2007). Bacteriocin-based strategies for food biopreservation. Int. J. Food Microbiol..

[6] Field D., Ross R.P., Hill C. (2018). Developing bacteriocins of lactic acid bacteria into next generation biopreservatives. Curr. Opin. Food Sci..

[7] Marsh K., Bugusu B. (2007). Food Packaging—Roles, Materials, and Environmental Issues. J. Food Sci..

[8] Cao N., Fu Y., He J. (2007). Preparation and physical properties of soy protein isolate and gelatin composite films. Food Hydrocoll..

[9] ASTM (2015). E 96/E96M–14: Standard Test Methods for Water Vapor Transmission of Materials.

[10] Gontard N., Duchez C., Cuq J.L., Guilbert S. (1994). Edible composite films of wheat gluten and lipids: Water vapour permeability and other physical properties. Int. J. Food Sci. Technol..

[11] ASTM (2014). D 882–12: Standard Test Method for Tensile Properties of Thin Plastic Sheeting.

[12] NCCLS (2013). Performance Standards for Antimicrobial Disk Susceptibility Tests; Approved Standard. NCCLS document M2-A8. Pennsylvania, USA. http://www.anvisa.gov.br/servicosaude/manuais/clsi/clsi_OPASM2-A8.pdf.

[13] Da Silva N., Junqueira V.C.A., de Arruda Silveira N.F., Taniwaki M.H., Gomes R.A.R., Okazaki M.M. (2012). Manual de Métodos de Análise Microbiológica de Alimentos e Água. (Manual of Microbiological Food and Water Analysis Methods).

[14] Riaz A., Lagnika C., Luo H., Dai Z., Nie M., Hashim M.M., Liu C., Song J., Li D. (2020). Chitosan-based biodegradable active food packaging film containing Chinese chive (*Allium tuberosum*) root extract for food application. Int. J. Biol. Macromol..

[15] Crizel T.M., Rios A.O., Alves V.D., Bandarra N., Moldão-Martins M., Flôres S.H. (2018). Active food packaging prepared with chitosan and olive pomace. Food Hydrocoll..

[16] Rhim J.W. (2011). Effect of clay contents on mechanical and water vapor barrier properties of agar-based nanocomposite films. Carbohydr. Polym..

[17] Casariego A., Souza BW S., Cerqueira M.A., Teixeira J.A., Cruz L., Díaz R., Vicente A.A. (2009). Chitosan/clay films’ properties as affected by biopolymer and clay micro/nanoparticles’ concentrations. Food Hydrocoll..

[18] Sothornvit R., Hong S.I., An D.J., Rhim J.W. (2010). Effect of clay content on the physical and antimicrobial properties of whey protein isolate/organo-clay composite films. LWT-Food Sci. Technol..

[19] Rhim J.W., Wang L.F. (2013). Mechanical and water barrier properties of agar/κ-carrageenan/konjac glucomannan ternary blend biohydrogel films. Carbohydr. Polym..

[20] Shankar S., Rhim J.W. (2016). Preparation of nanocellulose from micro-crystalline cellulose: The effect on the performance and properties of agar-based composite films. Carbohydr. Polym..

[21] Shahbazi Y., Shavisi N., Mohebi E. (2015). Effects of Ziziphora clinopodioides Essential Oil and Nisin, Both Separately and in Combination, to Extend Shelf Life and Control Escherichia coli O157:H7 and Staphylococcus aureus in Raw Beef Patty during Refrigerated Storage. J. Food Saf..

[22] Sobrino-López A., Martín-Belloso O. (2008). Use of nisin and other bacteriocins for preservation of dairy products. Int. Dairy J..

[23] Meira S.M.M., Zehetmeyer G., Werner J.O., Brandelli A. (2017). A novel active packaging material based on starch-halloysite nanocomposites incorporating antimicrobial peptides. Food Hydrocoll..

[24] Santiago-Silva P., Soares N.F.F., Nóbrega J.E., Junior M.A.W., Barbosa K.B.F., Volp A.C.P., Zerdas E.R.M.A., Wurlitzer N.J. (2009). Antimicrobial efficiency of film incorporated with pediocin (ALTA® 2351) on preservation of sliced ham. Food Control.

[25] Xiong Y., Chen M., Warner R.D., Fang Z. (2020). Incorporating nisin and grape seed extract in chitosan-gelatine edible coating and its effect on cold storage of fresh pork. Food Control.

[26] Deegan L.H., Cotter P.D., Hill C., Ross P. (2006). Bacteriocins: Biological tools for bio-preservation and shelf-life extension. Int. Dairy J..

[27] Jay J.M. (2005). Microbiologia de Alimentos.

[28] Botelho J., Araújo L.P.P., Pereira J.P.F., Taveira L.B., Furtado M.A.M., Pinto M.A.O. (2010). Microbiological quality of milk products rated by laboratory analysis of food and water from the Faculty of Pharmacy/UFJF. Rev. Inst. Laticínios Cândido Tostes.

[29] Lancette G.A., Bennett R.W., Downes F.P., Ito K. (2001). Staphylococcus aureus and staphylococcal enterotoxins. Compendium of Methods for the Microbiological Examination of Foods.

[30] Abdollahzadeh E., Hosseini H.M., Fooladi A.A.I. (2018). Antibacterial activity of agar-based films containing nisin, cinnamon EO, and ZnO nanoparticles. J. Food Saf..

[31] Capelezzo A.P., Mohr L.C., Dalcanton F., Barreta C.R.D.M., Martins M.A.P.M., Fiori M.A., Mello J.M.M. (2018). Antimicrobial biodegradable polymer through additivation with zinc based compounds. QUÍMICA NOVA.

[32] Hennekinne J.A., Buyser M.L., Dragacci S. (2012). Staphylococcus aureus and its food poisoning toxins: Characterization and outbreak investigation. FEMS Microbiol. Rev..

